# Mapping love: a personality-centered network analysis of relationship satisfaction

**DOI:** 10.3389/fpsyg.2025.1587405

**Published:** 2025-07-21

**Authors:** Oliver Tobias Schulz, Danièle Anne Gubler, Ursina Elsa Raemy, Stefan Johannes Troche

**Affiliations:** Department of Personality Psychology, Differential Psychology, and Assessment, Institute of Psychology, University of Bern, Bern, Switzerland

**Keywords:** relationship satisfaction, attachment, jealousy, self-esteem, relationship self-efficacy, sociosexuality, network analysis

## Abstract

Previous research has linked various personality features to relationship satisfaction, primarily investigating bivariate effects. Given the interrelatedness of these personality features, their unique associations with relationship satisfaction remain unclear. The present study addresses this gap by exploring the holistic interplay of relationship satisfaction with related personality features and considering gender as a moderator. With an online self-report survey, relationship satisfaction, attachment, jealousy and trust, self-esteem, relationship self-efficacy, sexual satisfaction, and sociosexuality in 510 women and 300 men (*M*_*age*_ = 26.5 years) were assessed. Network analysis was used to estimate a combined network, while a network comparison test was used to examine gender differences. Insecure attachment, trust, mutuality, and sexual satisfaction uniquely correlated with relationship satisfaction within the combined network. Networks of men and women were largely similar. These results expand the understanding of relationship satisfaction and inform the ongoing debate on gender differences in psychological research.

## Introduction

Satisfaction with romantic relationships is associated with higher levels of physical and psychological wellbeing (Dush and Amato, 2005; Proulx et al., 2007) and overall life satisfaction (Be et al., 2013; Yam, 2023). Contrarily, less satisfying relationships are relatively likely to dissolve (Gottman and Levenson, 1992). In the context of marriage, this often results in divorce, a phenomenon that has reached historical highs in recent decades (Ortiz-Ospina and Roser, 2020). Divorces not only impose financial burdens and strain on legal systems, but children of divorced parents also carry comparatively high risks of developing psychological disorders (Sands et al., 2017; Schaan et al., 2019). Addressing these far-reaching issues necessitates understanding what constitutes relationship satisfaction (RSA) and why some individuals are less satisfied with their relationships compared to others. Such knowledge can further help to maximize the benefits of maintaining satisfying relationships.

The degree to which someone is satisfied with a romantic relationship depends on a variety of factors, ranging from societal norms to the presence of children, the frequency, intensity, and severity of conflicts, and experiences of infidelity (Bradbury et al., 2000). Moreover, the extent to which worldviews, personalities, and preferences of individuals are compatible can also affect the quality of relationships (Huston and Houts, 1998). Personality and its relevance to romantic relationships has already drawn considerable scientific interest. Previous investigations have identified various personality features that are associated with RSA, including but not limited to, attachment (Stackert and Bursik, 2003), jealousy (Andersen et al., 1995) and trust (Fitzpatrick and Lafontaine, 2017), self-esteem (Fincham and Bradbury, 1993), relationship self-efficacy (Weiser and Weigel, 2016), sexual satisfaction (Byers, 2005), and sociosexuality (Penke and Asendorpf, 2008). Most studies have focused on bivariate effects, examining how RSA is linked to one feature at a time. However, given the substantial interrelatedness of the mentioned personality features, their unique associations with RSA remain unclear. The present study addresses this gap by exploring a more holistic interplay of RSA with relevant personality features, highlighting their unique associations, and considering gender as a moderator. Its focus lies on heterosexual men and women in monogamous relationships. In the following sections, we will first elaborate on how RSA is defined and measured. Then, we will focus on a selection of personality features linked to RSA and discuss potential gender differences in this context.

### Relationship satisfaction

RSA refers to a subjective evaluation of an interpersonal (here: romantic) relationship and answers the question of how someone feels about their relationship at a particular moment in time (Hendrick et al., 1988). Various approaches have been developed to assess RSA. For example, Spanier (1976) proposed that it can be determined by the frequency of affectionate behaviors (e.g., laughing together, kissing) and by the number of domains over which disagreements occur (e.g., household tasks, religious matters). Siffert and Bodenmann (2010) similarly argued that RSA encompasses multiple domains, such as mutual admiration, trust, and sexual satisfaction. In contrast, Fincham and Bradbury (1987) suggested that RSA should be captured as a single global entity, based on how well a relationship fulfills needs or meets expectations (Hendrick, 1988).

Some studies suggest that women experience lower levels of RSA than men (Lesch and Engelbrecht, 2011; Jackson et al., 2014). However, this discrepancy may be primarily attributed to unique sample characteristics (e.g., clinical samples or very specific cultural contexts). Overall, most studies report that levels of RSA are equal among men and women (Erol and Orth, 2014; Fallis et al., 2016).

### Attachment

Attachment is arguably the most basic psychological concept that underlies human bonding. Originating from Bowlby's (1969) theory about the development of attachment during infancy, several approaches to attachment in adult relationships exist today (Ravitz et al., 2010). For instance, Brennan et al. (1991) proposed that adult attachment can largely be reflected by two dimensions: *anxiety* and *avoidance*. Anxiety refers to the degree of concern individuals experience regarding rejection or abandonment. Avoidance refers to the extent to which individuals are uncomfortable with intimacy and to their preference to maintain emotional distance. Individuals can endorse any combination of anxiety and avoidance levels. Securely attached individuals usually endorse neither anxious nor avoidant attachment.

Insecurely (i.e., anxiously, avoidantly) attached individuals often experience lower levels of RSA, compared to securely attached individuals (Stackert and Bursik, 2003; Hirschberger et al., 2009). Li and Chan (2012) argue that attachment avoidance is particularly detrimental to RSA. While high levels of attachment anxiety are often associated with frequent conflicts, their negative effect on RSA may be buffered by the enjoyment that anxiously attached individuals experience in times when their relationships feel safe (Li and Chan, 2012). Contrarily, avoidantly attached individuals usually seek independence in their lives, thereby avoiding actions that could improve their RSA, such as openly discussing personal boundaries with their partner. As a result, they tend to maintain higher levels of dissatisfaction (Li and Chan, 2012).

Some studies suggest that genders are largely similar regarding adult attachment (Stackert and Bursik, 2003; Bakermans-Kranenburg and Ijzendoorn, 2009). However, other studies revealed that women more frequently endorse anxious attachment, while men are more likely to endorse avoidant attachment (Bartholomew and Horowitz, 1991; Butzer and Campbell, 2008). Moreover, gender has been shown to moderate the relationship between attachment and RSA. Barry et al. (2015) found that the negative effect of avoidant attachment on RSA is stronger in women than in men, while the negative effect of anxious attachment is stronger in men than in women.

### Romantic jealousy and trust

Romantic jealousy refers to the experience that arises following a perceived or actual threat to a romantic relationship (White, 1981). Experiencing romantic jealousy entails an aversive emotional state, while certain cognitions (e.g., worries about potential or actual extradyadic interest in a partner) and behaviors (e.g., surveillance activities or verbal aggression toward potential rivals) are also induced (Wegner et al., 2018). Although romantic jealousy can originate from a desire to protect a relationship and, thus, potentially enhance RSA (Rydell et al., 2004), it is more frequently negatively associated with RSA, ranging from depression to physical violence (Pines and Aronson, 1983; Andersen et al., 1995).

Meta-analyses on gender differences in romantic jealousy yielded inconclusive results (Harris, 2003; Carpenter, 2012; Sagarin et al., 2012). On the one hand, men tend to display larger discrepancies in sexual and emotional jealousy than women do (Edlund and Sagarin, 2017). On the other hand, gender differences can disappear when controlling for the type of response format in measurement tools (i.e., forced-choice or continuous scales; Carpenter, 2012). Thus, detecting gender differences in romantic jealousy might depend on aspects such as measurement technique, statistical methodology, and the inclusion of moderator variables like relationship status, attachment, and sexual orientation (Edlund and Sagarin, 2017).

Romantic jealousy is closely linked to trust, such that higher levels of jealousy coincide with lower levels of trust (Marshall et al., 2013; Rodriguez et al., 2015). Rempel et al. (1985) proposed that trust involves multiple elements, such as regarding a partner as reliable and helpful, believing that they care, and feeling confident in the relationship's strength. Existing evidence suggests that trust is positively correlated with RSA (Wieselquist, 2009; Fitzpatrick and Lafontaine, 2017). Furthermore, studies on gender differences have repeatedly shown that men and women share similar levels of trust within romantic relationships (Marshall et al., 2013; Fitzpatrick and Lafontaine, 2017; Yilmaz et al., 2023).

### Self-esteem

Self-esteem is an overall evaluation of oneself and can be regarded as a global judgment of the extent to which individuals perceive themselves as competent, worthy, and deserving of respect (Rosenberg, 1965). The sociometer theory posits that self-esteem is largely affected by social feedback (Leary and Baumeister, 2000). Receiving attention and appreciation, especially by a romantic partner, might increase levels of self-esteem or protect them from deterioration (Murray et al., 2003). Hence, self-esteem is positively linked to RSA (Fincham and Bradbury, 1993; Shackelford, 2001).

Gender differences in self-esteem appear to be relatively pronounced. Bleidorn et al. (2016) found that men consistently report higher levels of self-esteem compared to women, with a small to medium-sized effect, controlling for age and cultural diversity. Other studies provided further support for this finding (Feingold, 1994; Robins et al., 2002).

### Relationship self-efficacy

Bandura (1977) defined self-efficacy as an individual's belief in their ability to successfully perform specific tasks or achieve desired goals. In the context of romantic relationships, self-efficacy describes confidence in the ability to shape and influence outcomes of romantic relationships (Cabeldue and Boswell, 2012). Strong self-efficacy beliefs facilitate the successful attainment of goals, which can evoke feelings of accomplishment and satisfaction (Bandura, 1977). Consistent with this, positive links between relationship self-efficacy and RSA have been reported (Fincham et al., 2000; Weiser and Weigel, 2016).

Riggio et al. (2011) found that women tend to endorse stronger relationship self-efficacy beliefs than men. Lopez et al. (2007) examined the construct in more detail and found women to score higher on the perceived ability to both provide and receive care and support from a partner as well as to constructively discuss important matters within a romantic relationship (*mutuality*), whereas men scored higher on the perceived ability to regulate negative feelings toward a partner, like frustration, disappointment, and anger (*emotional control*). They observed no gender differences in *differentiation* referring to the perceived ability to express needs for separateness and to assertively maintain interpersonal boundaries (Lopez et al., 2007).

### Sexual satisfaction

Sexual satisfaction can be defined as “an affective response arising from one's subjective evaluation of the positive and negative dimensions associated with one's sexual relationship” (Lawrance and Byers, 1995, p. 268). Sexual satisfaction is positively linked to RSA and compared to other features relevant to RSA, it exhibits some of the highest correlations (Sprecher, 2002; Byers, 2005; Fallis et al., 2016). It is not clear whether sexual satisfaction should be considered as an independent feature or as a component of RSA (Hassebrauck and Fehr, 2002; Siffert and Bodenmann, 2010). Nevertheless, the consideration of sexual satisfaction seems to be mandatory when investigating individual differences in RSA.

Most studies suggest that men and women are equally satisfied with sexuality in their romantic relationships (Butzer and Campbell, 2008; Peixoto, 2023). Interestingly, sexual satisfaction seems to be a stronger predictor of subsequent RSA for men, compared to women (Hassebrauck and Fehr, 2002; Sprecher, 2002), although the opposite has also been observed (Vohs et al., 2004).

### Sociosexuality

Kinsey et al. (1948) originally described sociosexuality as a predisposition or willingness to engage in uncommitted sexual relationships. Individuals with an unrestricted sociosexual orientation are usually comfortable with casual sex, without feeling the need for emotional closeness or intimacy, with the opposite being true for individuals with a more restricted sociosexual orientation (Simpson and Gangestad, 1991). Penke and Asendorpf (2008) identified three components of sociosexuality. *Sociosexual behavior* represents the number of short-term sexual encounters an individual has had in the past as well as the tendency with which they will do so in the future. *Sociosexual attitude* entails personal opinions and broad evaluations regarding uncommitted sex. Finally, *sociosexual desire* refers to the degree to which an individual currently wishes to engage in uncommitted sexual activities. High levels of sociosexual desire can negatively affect RSA (Penke and Asendorpf, 2008). Moreover, high levels of sociosexual desire in both partners have been shown to predict relationship dissolution (Penke and Asendorpf, 2008). Finally, individuals with an unrestricted sociosexual orientation are more likely to engage in infidelity behaviors than individuals with a restricted sociosexual orientation (Mattingly et al., 2011).

While men and women tend to exhibit similar levels of sociosexual behavior, moderate differences in sociosexual attitude and large differences in sociosexual desire have been observed, with men scoring higher on both dimensions (Penke and Asendorpf, 2008). Moreover, men consistently report a more unrestricted global sociosexual orientation than women do (Schmitt, 2005; Sprecher et al., 2013). Furthermore, Webster et al. (2015) found that a negative correlation between sociosexuality and RSA exists for men, but not for women.

### The present study

The previous sections have explored the importance of various personality features in romantic relationships and their individual links to RSA. These features stem from different perspectives on personality, such as the social-cognitive (e.g., relationship self-efficacy), the attachment (e.g., avoidance), the trait-based (e.g., jealousy), or the evolutionary approach to human personality (e.g., sociosexual behavior). Therefore, it is not surprising that they are interconnected (e.g., Foster et al., 2007; Gubler et al., 2023; Richter et al., 2022; Riggio et al., 2013), and it is to be expected that they are jointly associated with RSA. Consequently, a holistic approach is necessary to better understand their complex interplay. A network approach is well-suited for this task. Unlike latent variable models or factor analyses, which often assume that observed variables are caused by underlying, unobserved traits, the network perspective proposes that personality features and other psychological phenomena are best understood as complex systems of interacting variables (Borsboom et al., 2021). Consequently, network analysis does not differentiate between independent and dependent variables but emphasizes the reciprocity of (correlational) relationships. It sees personality as an ecosystem of characteristics with stimulating and inhibitory relationships and shifts the focus from shared variance to the direct, unique relationships between observable variables (Costantini et al., 2015). Furthermore, by using partial correlations, the redundancy of different personality features (from the same or different approaches described above) in their association with RSA is explicitly addressed and revealed. These insights are typically lost when variables are aggregated in latent models and factor analyses. Network analysis also offers its own measures (e.g., centrality indices) to characterize the structure and organization of a network. These structural properties can yield important insights into the functioning of the whole system and the roles of individual variables within it. For example, *strength centrality* indicates the number of connections of a variable to all other variables, *closeness centrality* reflects the distance of a variable to all others, and *betweenness* identifies variables that frequently act as bridges between other variables (Costantini et al., 2015). Network analysis has repeatedly been used to investigate personality-related phenomena and is gaining increasing popularity (Costantini et al., 2019; Herzberg and Wildfang, 2018; Nickull et al., 2022). Accordingly, the first aim of this study is to illuminate if and how RSA is connected to attachment, jealousy and trust, self-esteem, relationship self-efficacy, sexual satisfaction, and sociosexuality within a network.

As outlined before, gender differences have been reported for most of the mentioned personality features, although some of this evidence is not consistent. The moderating role of gender in the associations between these personality features and RSA has also been investigated, albeit much less extensively. For example, Nickull et al. (2022) investigated the role of gender in the interplay between sexual satisfaction and RSA. They found that networks of men and women are largely similar, except that sexual satisfaction plays the most central role for men, while sexual desire holds that position for women (Nickull et al., 2022). Personality features were not considered in their study. Thus, to help understand the role of gender in the interplay between personality and RSA, the second aim of this study will be to investigate if and how men and women vary in their respective network structures.

## Materials and methods

We report all measures and exclusions in this study. Data and R code are available under https://osf.io/38qrt/?view_only=2e2e5b4c7b864eeface66b8436dce80b.

### Participants

Social media platforms, private group chats, and the university's participant pool were used to recruit participants. As a reward, five shopping vouchers worth CHF 20 were raffled off among participants. Psychology students enrolled at the University of Bern received 0.5 course credits in exchange for participation but were not included in the raffle. All participants provided their written informed consent by pressing a confirmation button in the online survey before participating. Participant recruitment started on October 1, 2022, and ended on January 31, 2024. The study was approved by the ethics committee of the Faculty of Human Sciences of the University of Bern prior to its launch (submission number 2022-03-00003). No preregistration was submitted for this exploratory research.

Overall, 2,180 individuals accessed our online survey programmed on Qualtrics, which contained questionnaires to measure the study variables and to collect information pertaining to romantic relationships (e.g., sexual orientation, experiences of infidelity) and sociodemographic characteristics. For this study, only data from individuals who self-reported as heterosexual men or women and who were in a committed, monogamous relationship at the time of participation were considered for analysis. Due to these criteria, data from 788 individuals were excluded. Data from a further 582 individuals were excluded due to an excess of 20% missing values. The final sample consisted of 810 individuals (510 female). The average age was 26.5 years (*SD* = 8.7 years), ranging from 18 to 80 years. The average relationship duration was 4.3 years (*SD* = 5.9 years). Thirty percent of participants reported that they had previously experienced some form of infidelity (i.e., emotional, sexual, other) within a romantic relationship. Participants were well-educated, with 52% possessing a university entrance certificate, and 34% a university degree.

To tackle the second research objective, two subgroups consisting of 300 men and 300 matched women were formed (see Statistical analyses). On average, men were 28.9 years old (*SD* = 9.6 years), while women had an average age of 27.6 years (*SD* = 8.6 years). The age difference between genders was not significant, *t*_(598)_ = 1.697, *p* = 0.090, Cohen's *d* = 0.139. The difference between the two groups in relationship duration with 5.1 years (*SD* = 6.8 years) for men and 4.5 years for women (*SD* = 6.4 years) was not statistically significant, *t*_(598)_ = 1.611, *p* = 0.306, *d* = 0.084. The female group (*M* = 3.93, *SD* = 1.09) differed significantly in educational level from the male group (*M* = 3.73, *SD* = 1.19) with $\chi_{\left(6\right)}^{2}$ = 14.178, *p* = 0.028, Cramér's *V* = 0.15. Finally, 30% of men and 36% of women reported having experienced infidelity within a romantic relationship. This difference was not significant, $\chi_{\left(1\right)}^{2}$ = 2.168, *p* = 0.141, Cramér's *V* = 0.05.

### Measures

#### Relationship satisfaction

The German version (Sander and Böcker, 1993) of the Relationship Assessment Scale (RAS; Hendrick, 1988) was used to assess RSA, which is suitable for both marital and non-marital partnerships (Renshaw et al., 2011). The scale consists of seven items (e.g., “How good is your relationship compared to others?”) to be rated on a five-point response scale ranging from *very poor* (1) to *very good* (5), with higher values indicating higher levels of RSA. The RAS shows high internal consistency (α = 0.89), and its factorial validity has been confirmed (Dinkel and Balck, 2005).

#### Attachment

The revised, eight-item version of the Experiences in Close Relationships questionnaire in German (ECR-RD8; Ehrenthal et al., 2021) was utilized to measure adult attachment style in romantic relationships. It comprises eight items, reflecting anxious or avoidant attachment tendencies, measured by four items each. Items (e.g., “I often worry that my partner will not want to stay with me.”) are rated on a seven-point response scale ranging from *strongly disagree* (1) to *strongly agree* (7), with higher scores indicating greater levels of attachment insecurity. The ECR-RD8 exhibits good reliability (McDonald's ω > 0.80 for both subscales) and convergent validity with other attachment scales (Ehrenthal et al., 2021).

Participants also completed the German version of the Adult Attachment Scale (Schmidt et al., 2004) as an additional measure of attachment style. However, due to its weaker psychometric properties compared to the ECR-RD8, data collected from this scale were excluded from our analysis.

#### Romantic jealousy and trust

Romantic jealousy and trust were both assessed using the German self-report questionnaire developed by Bauer (1988). Therein, jealousy is measured with 10 items (e.g., “I always want to know what my partner is doing when he/she is not with me.”) while trust is measured with five items (e.g., “I believe that my partner is absolutely open with me.”). Items are rated on a six-point response scale ranging from *does not apply at all* (1) to *applies exactly* (6), with higher scores indicating higher levels of jealousy and trust. The scale shows good reliability (α = 0.87) and convergent validity with general jealousy tendencies (Schmitt et al., 1995).

#### Self-esteem

Self-esteem was assessed with the German translation (Ferring and Filipp, 1996) of the Rosenberg Self-Esteem Scale (RSES; Rosenberg, 1965). The RSES consists of 10 items (e.g., “Overall, I am satisfied with myself”). Items are rated on a four-point response scale ranging from *does not apply at all* (1) to *applies exactly* (4), with higher values indicating higher levels of self-esteem. The German scale exhibits good internal consistency (α = 0.84; Collani and Herzberg, 2003).

#### Relationship self-efficacy

For this study, the English language Relationship Self-Efficacy Scale (Lopez et al., 2007) was translated into German following the guidelines of the International Test Commission (http://www.intestcom.org) and with the consent of the test author, Frederick G. Lopez. The questionnaire consists of 25 items measuring confidence in the ability to contribute to the success of a relationship. Relationship self-efficacy is underpinned by three latent factors: mutuality (16 items, e.g., “How confident are you in your ability to openly and directly address significant disagreements?”), emotional control (four items, e.g., “How confident are you in your ability to stay calm when you and your partner are having a serious argument?”), and differentiation (five items, e.g., “How confident are you in your ability to tell your partner when you need to be alone?”). Items are rated on a nine-point response scale, ranging from *not at all confident* (1) to *very confident* (9), with higher scores indicating higher levels of relationship self-efficacy. The English version of the questionnaire demonstrates very good (α = 0.94) internal consistency and construct validity was supported by significant correlations with attachment components and RSA (Lopez et al., 2007).

#### Sexual satisfaction

Sexual satisfaction was assessed with the subscale *Sexuality in the Relationship* from the *Partnership Quality Questionnaire* (FPQ; Siffert and Bodenmann, 2010). The subscale consists of five items (e.g., “Our partnership is sexually satisfying to me.”), each rated on a five-point response scale ranging from *strongly disagree* (1) to *strongly agree* (5). Higher scores indicate higher levels of sexual satisfaction. The subscale shows very high internal consistency (α = 0.94) and convergent validity with all subscales correlating with the RAS (Siffert and Bodenmann, 2010). In our study, all subscales of the FPQ were presented to participants. However, only the sexual satisfaction subscale was included in our analysis, as most constructs represented by the other subscales were already considered in the network (e.g., trust).

#### Sociosexuality

To measure sociosexuality, the revised Sociosexual Orientation Inventory (SOI-R; Penke and Asendorpf, 2008) was employed. The questionnaire assesses sociosexual behavior (e.g., “With how many partners have you had sex within the past 12 months?”), sociosexual attitude (e.g., “Sex without love is OK”), and sociosexual desire (e.g., “In everyday life, how often do you have spontaneous fantasies about having sex with someone you have just met?”). Each subscale is measured by three items, captured on a nine-point response scale with varying labels. Higher scores indicate a more unrestricted sociosexual orientation. Internal consistency of the subscales ranges from α = 0.76 to α = 0.88 (Penke, 2011).

### Statistical analyses

Network analysis was conducted to tackle both research objectives. It is suitable to investigate statistical relationships among multiple variables by visualizing the underlying partial correlation matrix in an easily interpretable manner (Costantini et al., 2015). Typically, networks consist of *nodes* representing the network variables and *edges* reflecting the statistical association among them. Centrality indices can be computed, which provide information about the importance of individual network variables (Costantini et al., 2015). Additionally, indirect bivariate effects can be inferred from networks, which can serve as a basis for investigating potential mediation effects (Epskamp and Fried, 2018).

All statistical analyses were conducted using R (version 4.3.1; Team, 2023). The analyzed data set did not include any missing values. For the upcoming sections, we followed the reporting guidelines laid out by Burger et al. (2023). As a first step, a mean score was computed for each subscale where applicable, resulting in 13 network nodes: RSA, attachment anxiety, attachment avoidance, jealousy, trust, self-esteem, mutuality, emotional control, differentiation, sexual satisfaction, sociosexual attitude, sociosexual desire, and sociosexual behavior. Despite their conceptual overlap, treating each subscale as a distinct node in the network aligns with the theoretical shift toward viewing psychological phenomena as complex systems of interacting variables (Borsboom et al., 2021). This approach allows for a more detailed examination of the direct relationships, complex structure, and specific components of the examined features.

Second, the *estimateNetwork* function from the *bootnet* package (version 1.5.6; Epskamp et al., 2018) was employed for network estimation. The Extended Bayesian Information Criterion was used to select the network that best fits the data (Chen and Chen, 2008). Using the graphical LASSO (glasso; Friedman et al., 2008), trivially small edges were removed. While default settings of the *bootnet* package were largely retained, the EBIC tuning parameter was set to 0.9. This value exceeds the commonly recommended range in psychological network research (Epskamp and Fried, 2018), reflecting a more conservative model selection approach. A higher tuning parameter imposes a stronger penalty on edge inclusion, resulting in a sparser network. This helps reduce the likelihood of false positives (i.e., spurious associations), particularly valuable in exploratory settings, and may enhance both the interpretability and replicability of the network structure. Notably, results using a lower tuning parameter (i.e., 0.5) produced a similar network structure, suggesting that the findings are robust to this analytic choice. The sum of all absolute edge weights was used to represent the global strength of the network. Visualization of the network was performed using the *qgraph* package (version 1.9.8; Epskamp et al., 2012) and the Fruchterman-Reingold algorithm (Fruchterman and Reingold, 1991). To assess the accuracy and stability of edge weights, 1,500 bootstrap samples were simulated.

Third, to investigate the relative importance of each node, *strength* and *closeness* centrality were examined (Costantini et al., 2015). Strength centrality reflects the total sum of all edge weights of a node and indicates how strongly the node is connected to all other nodes in the network. Closeness centrality quantifies how well-connected a node is, that is, to what extent a node reciprocally stimulates or inhibits surrounding nodes (Costantini et al., 2015). To assess the accuracy of centrality indices, we used case-drop bootstrapping based on 1500 samples (Epskamp et al., 2018). The resulting Correlation Stability coefficient (CS coefficient) quantifies the rank order stability of centrality measures using stepwise reduction of the sample. The CS coefficient measures the proportion of the sample that can be omitted while ensuring that the correlation between the rank order of centrality measures in the reduced sample and the original sample remains at least 0.7, with a 95% probability. To ensure that centrality measures remain interpretable, CS coefficients should be at least 0.25, ideally 0.50 (Epskamp et al., 2018).

Finally, to compare networks between women and men, two groups were formed. RSA is susceptible to temporal changes and common covariates include age and relationship duration (Erol and Orth, 2014). Hence, participants were assigned so that both groups showed similar levels in these variables. The *MatchIt* package (version 4.5.5; Ho et al., 2007) was used for this purpose. To achieve identical sample sizes for each group, the data from all men (*n* = 300) were used, along with data from the 300 women who best matched these men in the specified criteria. To compare networks for men and for women, the *NCT* function from the *NetworkComparisonTest* package (version 2.2.2) was employed (van Borkulo et al., 2023), using 2000 iterations.

## Results

Descriptive statistics for each network variable are presented in Table 1. Values of skewness and kurtosis show that most network variables follow an approximate normal distribution. Departure from normality (absolute skewness >2 or absolute kurtosis >4; Kim, 2013) in trust was deemed unproblematic due to a sufficiently large sample size (Sainani, 2012). The Supplementary material contains a table presenting the zero-order correlations of all study variables.

**Table 1:** Descriptive statistics and intercorrelations of relationship satisfaction and related personality features (*N* = 810).

**Network variable**	***M***	***SD***	**Skewness**	**Kurtosis**	**α**	**Zero-order correlation with RSA**	**Partial correlation with RSA**
Relationship satisfaction	4.37	0.54	−1.17	1.29	0.84	–	–
Attachment anxiety	2.32	1.29	1.13	0.74	0.76	−0.46^*^	−0.17
Attachment avoidance	1.84	0.85	1.36	2.13	0.69	−0.60^*^	−0.22
Jealousy	2.41	0.93	1.13	1.26	0.88	−0.19^*^	0.01
Trust	5.33	0.82	−2.55	8.99	0.86	0.39^*^	0.10
Self-esteem	3.20	0.58	−0.78	0.08	0.90	0.30^*^	0.03
Mutuality	7.62	0.99	−1.08	1.48	0.90	0.67^*^	0.24
Emotional control	6.45	1.50	−0.47	−0.07	0.82	0.42^*^	0.04
Differentiation	6.77	1.40	−0.79	0.47	0.82	0.54^*^	0.07
Sexual satisfaction	4.09	0.85	−0.91	0.41	0.89	0.43^*^	0.22
Sociosexual attitude	5.96	2.31	−0.50	−0.78	0.83	−0.07	0.00
Sociosexual desire	2.80	1.60	1.15	0.78	0.85	−0.20^*^	−0.01
Sociosexual behavior	2.55	1.49	1.28	0.85	0.73	−0.14^*^	−0.05

Values of partial correlations were not tested for statistical significance due to issues associated with family-wise error rates (Epskamp and Fried, 2018). ^*^p < 0.05.

### General network structure

**Figure 1:**
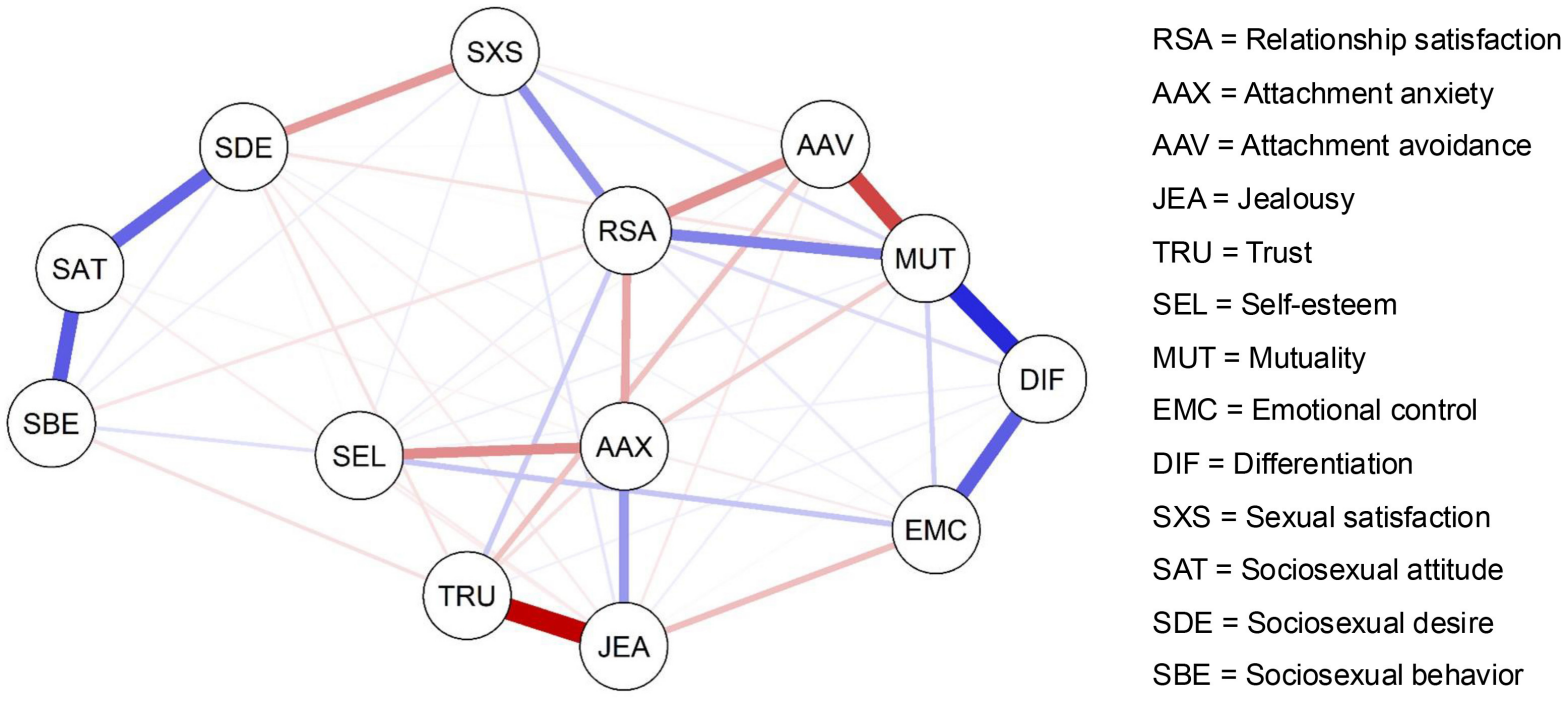
Estimated network structure of relationship satisfaction and related personality features (*N* = 810). Blue bars represent positive edges between nodes, whereas red bars represent negative edges. Line thickness reflects effect size, with thicker edges reflecting stronger partial correlations.

Figure 1 depicts the estimated network structure of 810 individuals. Out of 78 possible edges, 52 edges remained substantial and were retained in the network, meaning that 26 edges were set to zero by the glasso algorithm (Friedman et al., 2008). Global network strength was 5.51. Edge weights ranged from *r* = −0.50 (jealousy–trust) to *r* = 0.42 (differentiation–mutuality).

To explore the position and edges of RSA within the network, zero-order Pearson correlations and partial correlations (i.e., edge weights) of RSA with all network variables were computed (see Table 1). Most partial correlations were much smaller compared to their corresponding zero-order correlations and, in many cases, approached zero. Nodes that formed substantial partial correlations with RSA included attachment anxiety (*r* = −0.17), attachment avoidance (*r* = −0.22), trust (*r* = 0.10), mutuality (*r* = 0.24), and sexual satisfaction (*r* = 0.22). These variables uniquely correlated with RSA, persisting beyond the confounding effect of other nodes. Contrarily, the unique associations of RSA with jealousy (*r* = 0.01), self-esteem (*r* = 0.03), emotional control (*r* = 0.04), differentiation (*r* = 0.07), sociosexual attitude (*r* = 0.00), sociosexual desire (*r* = −0.01), and sociosexual behavior (*r* = −0.05) became negligible after controlling for the confounding effect of other nodes.

**Figure 2:**
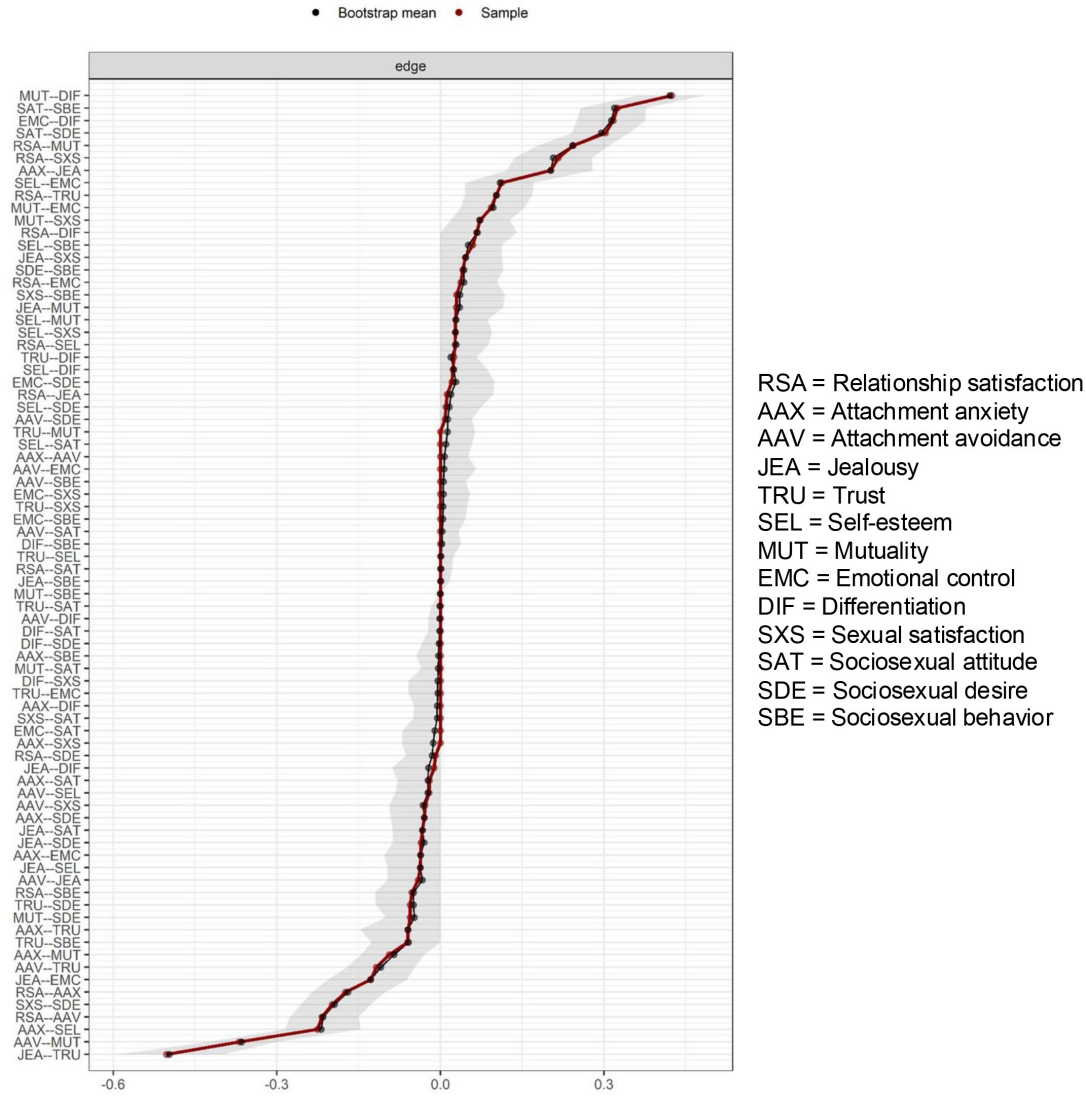
Bootstrapped accuracy test on 78 edge weights with 95% confidence intervals (*N* = 810). Each of the 78 edge weights is represented by both a black dot and a red dot. Black dots indicate average edge weights based on 1,500 bootstrap samples. Red dots indicate estimated edge weights in the study sample. Gray areas represent 95% confidence intervals for each value.

Figure 2 presents the bootstrapped confidence intervals and estimated values of all 78 edge weights. Confidence intervals were relatively small, indicating high robustness of the estimated edge weights. Note that confidence intervals in this context do not provide any information regarding null hypothesis testing, due to problems associated with family-wise error rates (Epskamp and Fried, 2018). In other words, if a confidence interval did not contain zero, this does not necessarily mean that the edge weight was significantly different from zero. Instead, confidence intervals should merely be regarded as indicators of the accuracy and stability of edge weights (Epskamp and Fried, 2018).

**Figure 3:**
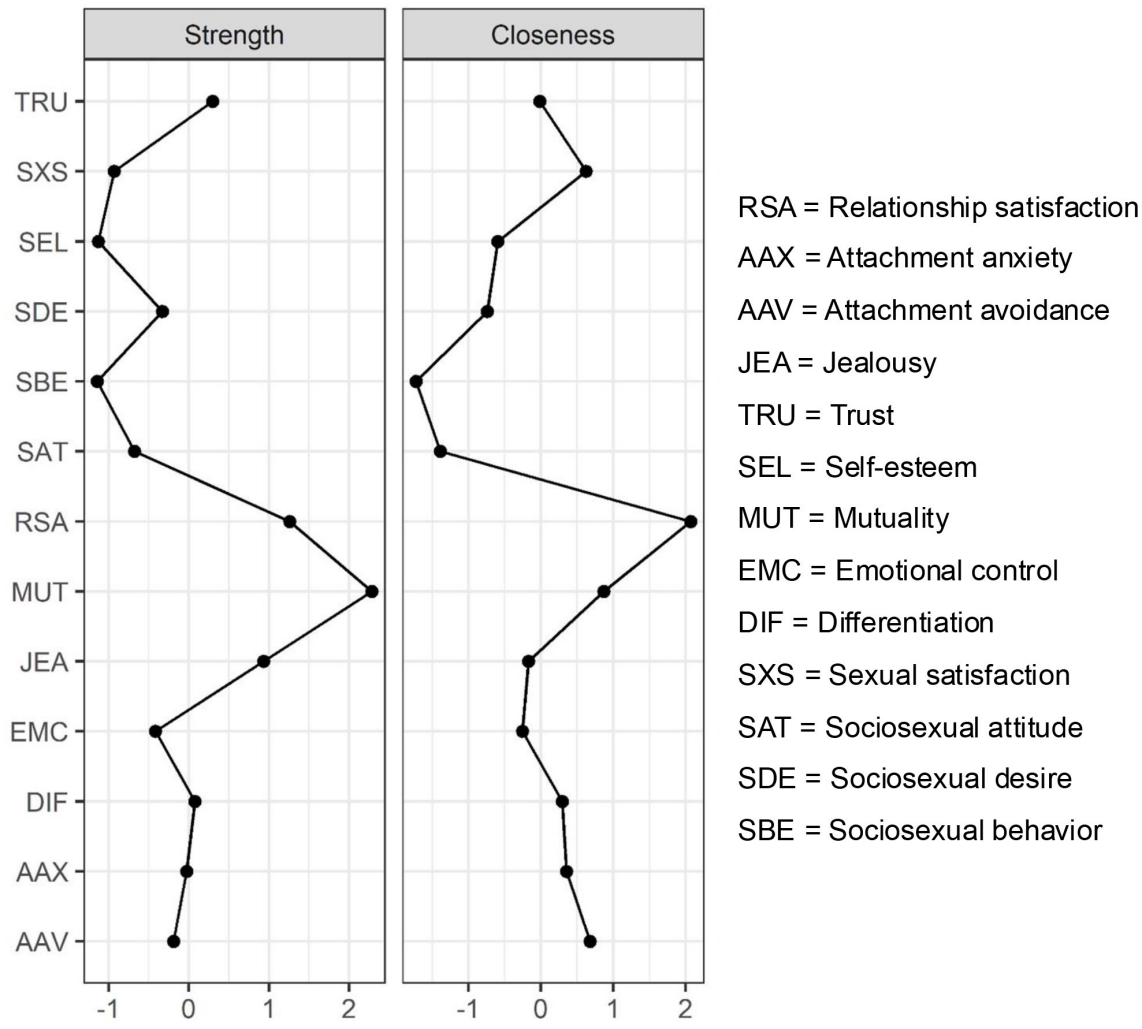
Estimated values for strength and closeness centrality of relationship satisfaction and related personality features (*N* = 810). For easier comparison of centrality indices, *z*-scores are displayed on the *x*-axis, rather than raw values.

Figure 3 presents the results of our centrality analyses. The robustness of both strength centrality (CS coefficient = 0.75) and closeness centrality (CS coefficient = 0.75) was high. RSA showed the highest closeness centrality (closeness = 2.07), and the second highest strength centrality (strength = 1.26). A similar finding surfaced for mutuality, exhibiting the highest strength centrality (strength = 2.28), and the second highest closeness centrality (closeness = 0.87).

### Network comparison

The second aim of this study was to compare network structures between heterosexual men and women. For valid comparisons of correlations (or edges in networks), scales should demonstrate measurement invariance across groups, at least on a metric level (Thompson, 2016). Therefore, invariance analyses were conducted for all subscales, using structural equation modeling (Thompson, 2016). The precondition of at least partial metric invariance of the subscales for women and men was given, as demonstrated in the Supplementary material. There, we also report where model modifications were necessary to obtain metric invariance.

Table 2 contains the results of group mean comparison tests for all network variables. Women and men did not significantly differ in RSA, attachment anxiety, attachment avoidance, trust, self-esteem, mutuality, differentiation, and sociosexual behavior. Women scored significantly higher than men on jealousy and sexual satisfaction, with small effect sizes each (Cohen, 1988). Men scored significantly higher than women on emotional control (medium effect), sociosexual attitude (small effect), and sociosexual desire (large effect).

**Table 2:** Means, standard deviations, and group comparisons by gender for relationship satisfaction and related personality features (*n* = 300 per group).

**Variable**	**Women**		**Men**		***t*_(598)_**	**Cohen's *d* (95% CI)**
	***M***	***SD***	***M***	***SD***		
Relationship satisfaction	4.34	0.59	4.37	0.53	−0.57	−0.05 (−0.21; 0.11)
Attachment anxiety	2.37	1.30	2.19	1.32	1.67	0.14 (−0.02; 0.30)
Attachment avoidance	1.80	0.92	1.90	0.86	−1.35	−0.11 (−0.27; 0.05)
Jealousy	2.45	0.86	2.25	1.00	2.62^**^	0.21 (0.05; 0.37)
Trust	5.36	0.71	5.30	1.03	0.74	0.06 (−0.10; 0.22)
Self-esteem	3.21	0.56	3.28	0.57	−1.59	−0.13 (−0.29; 0.03)
Mutuality	7.67	1.04	7.52	1.02	1.76	0.14 (−0.02; 0.30)
Emotional control	6.14	1.50	6.96	1.40	−6.88^***^	−0.56 (−0.73; −0.40)
Differentiation	6.80	1.44	6.78	1.45	0.21	0.02 (−0.14; 0.18)
Sexual satisfaction	4.18	0.82	4.01	0.89	2.53^*^	0.21 (0.05; 0.37)
Sociosexual attitude^a^	5.70	2.42	6.58	2.12	−4.72^***^	−0.39 (−0.55; −0.22)
Sociosexual desire^a^	2.30	1.10	3.71	1.84	−11.36^***^	−0.93 (−1.10; −0.76)
Sociosexual behavior	2.73	1.56	2.88	1.64	−1.12	−0.09 (−0.25; 0.07)

^a^Assumption of homogeneity of variance was violated, as indicated by Levene's test, *p* < 0.05. For these variables, a Welch correction was applied to the *t*-test results.

^*^p < 0.05, ^**^p < 0.01, and ^***^p < 0.001.

**Figure 4:**
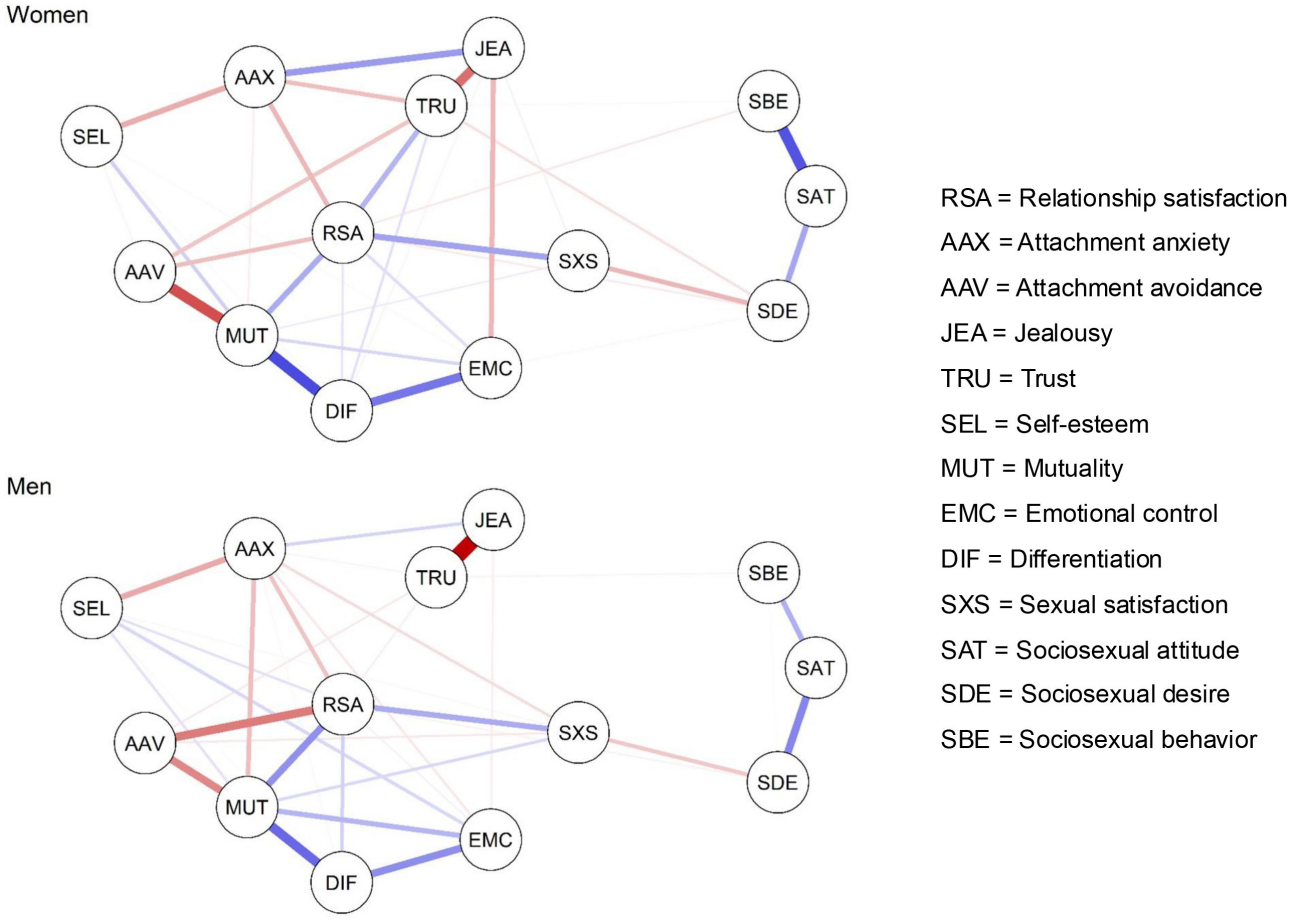
Estimated network structure of relationship satisfaction and related personality features for women (*n* = 300) and men (*n* = 300). Blue bars represent positive edges between nodes, whereas red bars represent negative edges. Line thickness reflects effect size, with thicker edges reflecting stronger partial correlations.

Figure 4 shows the estimated network structures for women and men, respectively. To compare networks, respective measures of global strength, network structure, and edge weight differences were computed. Concerning global strength, the NCT revealed no significant gender difference (difference in global strength = 0.21, *p* = 0.705). Thus, the total sum of edge weights was approximately equal in both networks. Next, a difference test between the two precision matrices yielded a statistically significant result (maximum edge difference = 0.27, *p* = 0.006), indicating that at least one edge weight significantly differed between the two networks.

To identify which edge(s) varied between networks, an edge weight difference test was performed. Six edge weights were significantly different between women and men: sociosexual behavior–sociosexual attitude (women: *r* = 0.40; men: *r* = 0.18; *p* = 0.001), jealousy–trust (women: *r* = −0.34; men: *r* = −0.59; *p* = 0.005), attachment anxiety–sexual satisfaction (women: *r* = 0.00; men: *r* = −0.08; *p* = 0.016), trust–differentiation (women: *r* = 0.07; men: *r* = 0.00; *p* = 0.025), RSA–trust (women: *r* = 0.17; men: *r* = 0.03; *p* = 0.023), and RSA–attachment avoidance (women: *r* = −0.15; men: *r* = −0.31; *p* = 0.027). However, after applying a Bonferroni correction for multiple tests, none of the observed differences remained significant, indicating that there were no substantial differences between the networks of men and women.

## Discussion

Previous research has identified various personality features that are linked to RSA but has primarily focused on bivariate effects. These features stem from different personality approaches and provide different perspectives on similar constructs. Due to this interrelatedness, it remains uncertain how each personality feature uniquely correlates with RSA. The present study sheds light on this uncertainty by exploring the interplay of RSA with its related personality features, using network analysis. The first aim was to determine the unique connections and the position of RSA within a network structure, consisting of attachment, jealousy and trust, self-esteem, relationship self-efficacy, sexual satisfaction, and sociosexuality. RSA and mutuality were the most central nodes in the network. Attachment, trust, mutuality, and sexual satisfaction were directly linked to RSA, whereas its links to jealousy, self-esteem, emotional control, differentiation, and sociosexuality became negligible. The second aim of this study was to explore the moderating role of gender. Networks of men and women were largely similar, implying that no substantial differences between genders exist in the interplay between RSA and personality.

RSA and mutuality were the most integral nodes in the network, as measured by strength and closeness centrality, thereby showcasing high potential to reciprocally activate or inhibit surrounding nodes (Costantini et al., 2015). This result supports the idea that RSA is intricately tied to a complex interplay of personality features that pertain to romantic relationships, with these features strongly depending on one another. Mutuality plays a similarly crucial role in this interplay, exhibiting a particularly strong connection to RSA.

Attachment anxiety and avoidance were negatively, trust, mutuality, and sexual satisfaction positively associated with RSA in the network, that is, after controlling for the effect of other relevant variables. This is in line with previous reports of positive correlations between RSA and trust (Fitzpatrick and Lafontaine, 2017), relationship self-efficacy (Weiser and Weigel, 2016), and sexual satisfaction (Byers, 2005), and with reports of negative correlations between RSA and insecure attachment (Stackert and Bursik, 2003). The present results not only confirm these previously reported associations but also reinforce their stance, as they appear to persist beyond the confounding effect of related variables.

The positive association between RSA and trust indicates that individuals who have faith in the strength of their relationship and who believe that their partner will provide help when needed, are generally more satisfied within the relationship. Similarly, individuals who are satisfied with sexual activities within the relationship are likely to experience higher levels of RSA. Moreover, mutuality emerged as a particularly important correlate of RSA, further supporting the finding that individuals with a conviction of being able to mutually provide and receive emotional support within a romantic relationship are concurrently more satisfied with it. Furthermore, anxious and avoidant attachment both correlate negatively with RSA. Individuals who either fear being abandoned or who are uncomfortable with emotional intimacy, tend to be less satisfied with their relationship, regardless of other features, such as trust or jealousy.

While some features formed substantial edges with RSA in the network, other features that have previously been shown to correlate with RSA took comparatively peripheral positions in the network, namely jealousy (Andersen et al., 1995), self-esteem (Fincham and Bradbury, 1993), and sociosexuality (Webster et al., 2015).

In our network, the link between self-esteem and RSA was primarily expressed through attachment anxiety, which was directly connected to both constructs and formed the shortest indirect path between them. This aligns with previous findings showing negative correlations between attachment anxiety and both self-esteem (Foster et al., 2007) and RSA (Stackert and Bursik, 2003). Although a direct edge between self-esteem and RSA was present, it was notably weak. This stands in contrast to much of the literature reporting stronger associations between the two (Fincham and Bradbury, 1993). Since network edges reflect associations that remain after controlling for other variables, this weak edge suggests that the relationship between self-esteem and RSA may be largely accounted for by shared connections with other variables, particularly attachment anxiety. Hence, individuals with low self-esteem are not necessarily dissatisfied with their relationships *per se*, but may experience heightened attachment anxiety, which in turn shapes the perception of their relationships. Furthermore, given the view that self-esteem comprises various domains (e.g., academic, physical, social; Shackelford, 2001), only its relational aspect may be meaningfully tied to RSA, which could explain the relatively modest direct association observed in the network.

Regarding jealousy, previous studies have found a negative correlation with RSA (Andersen et al., 1995), as confirmed by our correlational analyses. Much like the link between RSA and self-esteem, this direct association was substantially reduced in the network, implying that jealousy and RSA mostly covary due to their respective associations with trust and attachment anxiety.

Finally, no substantial edges were present among facets of sociosexuality and RSA in the network. What these features share in variance was mainly captured by sexual satisfaction. More specifically, sociosexual desire and sexual satisfaction formed a substantial edge, as well as sexual satisfaction and RSA. This pattern aligns with previous findings, showcasing a positive association between sexual satisfaction and RSA (Butzer and Campbell, 2008).

Taken together, when looking at the interplay of RSA and personality features holistically, some features play a more prominent role (trust, mutuality, sexual satisfaction, anxious, and avoidant attachment), whereas others have a less profound impact (jealousy, self-esteem, sociosexuality, emotional control, and differentiation). These insights only emerge once the interplay between personality and RSA is viewed from a holistic, comprehensive perspective, and by taking many relevant variables into account.

The second aim of this study was to investigate whether and how gender affects the network structure of RSA and its corresponding personality features. A large body of research has investigated gender differences in personality features, yielding both robust and inconclusive findings. Furthermore, evidence regarding the moderating effect of gender on the associations between personality and RSA is scarce, and synthesizing existing literature into a conclusive picture remains challenging. To address this issue, additional network analyses were conducted separately for men and women.

Regarding mean differences, men scored higher on emotional control as well as sociosexual attitude and desire than women. Women scored higher than men on jealousy and sexual satisfaction. Finally, no differences were found in RSA, attachment anxiety and avoidance, trust, self-esteem, mutuality, differentiation, and sociosexual behavior. However, more relevant to the present purpose was the fact that the associations between those features did not differ between men and women, despite some differences in means.

Networks of men and women were highly similar in this study. The findings align with the gender similarity hypothesis (Hyde, 2005) and the notion that within-gender variation often exceeds between-gender variation (Feingold, 1994; Schmitt, 2005). Some gender differences emerged, particularly in the edges between RSA and trust, as well as avoidant attachment. Although these differences did not remain significant after Bonferroni correction, small but true effects may have been obscured due to this rather conservative method. Such strict corrections are used to avoid type I errors, but this increases the risk of failing to detect true differences and increases type II errors. Thus, while the overall pattern supports the idea that men and women are more similar than different in how personality and RSA are connected, this conclusion should be drawn with caution. Accordingly, the network interpretation for the total sample appears broadly applicable to both men and women, though subtle differences may exist.

## Limitations and future research

Self-report measures on sensitive topics such as sexual satisfaction and jealousy may be subject to social desirability bias, potentially leading participants to underreport negative emotions or agree more strongly with socially accepted statements. This could affect the accuracy of the observed associations in the network. Prior research has shown that individuals often adjust their responses on intimate topics to align with perceived social norms or to maintain a positive self-image (Tourangeau and Yan, 2007). While anonymity was ensured to mitigate such biases, their influence cannot be entirely ruled out.

Despite the wide age range of participants in this study, the average age was relatively low. This may limit the extent to which the findings apply to older individuals, and caution is warranted when interpreting the results across different age groups.

A *post-hoc* power analysis, conducted with the R package *powerly* (version 1.8.6; Constantin et al., 2023), revealed that at least 1,904 participants would be required to achieve a statistical power of 0.8 while maintaining similar network characteristics (nodes = 13; density = 0.67; coefficient range = −0.6 to 0.5). However, this estimation requires an a priori definition of the network structure. Due to the exploratory nature of the present study, no such definition was formulated and according to the conducted stability tests, the sample size appeared sufficiently large to yield robust results for the combined network (Epskamp and Fried, 2018).

Our network contained only undirected effects; hence, causal pathways were not explored. Directed hypotheses could be examined in future investigations, but would require longitudinal data (Borsboom et al., 2021). Applying such an approach to the present study variables would help better understand the meaning of the relationships identified in the present study.

Another avenue for future research involves the investigation of partner effects. The results of the present study imply that similarities rather than differences in personality features between romantic partners might be slightly more purposeful if the goal is to maintain a satisfying relationship. Yet, it has not been specifically investigated if individuals who are satisfied with their relationships exhibit similar levels of relevant personality features as their romantic partners. Focusing on network comparisons on a dyadic level could further contribute to understanding individual differences in RSA.

Finally, this study demonstrated that mutuality plays a key role within the relational network. To improve RSA, one practical avenue may lie in enhancing self-efficacy beliefs, specifically related to mutuality. Evidence suggests that self-efficacy beliefs are susceptible to training interventions (Vîslǎ et al., 2022). Therapeutic interventions, particularly couples therapy, can provide a structured and safe environment in which partners can develop and strengthen mutual behaviors such as emotional attunement, responsiveness, and collaborative problem-solving (Roddy et al., 2020). As couples gain mastery in these areas, their confidence in their ability to co-create a supportive and reciprocal relationship is likely to increase, thereby reinforcing mutuality and, potentially, enhancing overall RSA (cf. Satir, 1976).

## Conclusion

In summary, the present study confirms most of the extant evidence regarding the role of personality in RSA, while also providing a more differentiated perspective on this complex interplay. Mutuality, a specific domain of relationship self-efficacy, appears to play a key role in maintaining satisfying relationships and could guide interventions that are designed to enhance satisfaction with romantic relationships. While men and women differ in a few aspects that constitute RSA, their respective networks are largely similar. Results contribute to the ongoing debate regarding gender differences in personality and generally support the notion that men and women share more similarities than differences when it comes to RSA and personality features that pertain to it.

## Data Availability

The datasets presented in this study can be found in online repositories. The names of the repository/repositories and accession number(s) can be found below: Data and R code are available under http://osf.io/wxgyd.
