# Supplementary material for: Mapping love: a personality-centered network analysis of relationship satisfaction
Source: Front Psychol. 2025 Jul 21;16:1587405. doi: 10.3389/fpsyg.2025.1587405 (PMC12318981; doi:10.3389/fpsyg.2025.1587405)
Supplement: Supplementary file 1 [file Supplementary_file_1.pdf]

Zero-Order Pearson Correlations

Table S1. Zero-Order Pearson Correlations of Study Variables.

	RSA	AAX	AAV	JEA	TRU	SEL	MUT	EMC	DIF	SXS	SAT	SDE
AAX	-0.46*											
AAV	-0.60*	0.34*										
JEA	-0.19*	0.42*	0.14*									
TRU	0.39*	-0.38*	-0.36*	-0.62*								
SEL	0.30*	-0.40*	-0.26*	-0.25*	0.20*							
MUT	0.67*	-0.44*	-0.68*	-0.17*	0.35*	0.32*						
EMC	0.42*	-0.35*	-0.31*	-0.33*	0.25*	0.32*	0.51*					
DIF	0.54*	-0.36*	-0.47*	-0.27*	0.32*	0.29*	0.72*	0.58*				
SXS	0.43*	-0.21*	-0.32*	0.00	0.16*	0.17*	0.38*	0.20*	0.23*			
SAT	-0.07	-0.06	0.08*	-0.08*	-0.04	0.06	-0.09*	-0.04	-0.05	-0.11*		
SDE	-0.20*	-0.01	0.18*	-0.05	-0.14*	0.02	-0.21*	-0.01	-0.13*	-0.30*	0.39*	
SBE	-0.14*	0.00	0.09*	0.03	-0.14*	0.08*	-0.07*	0.00	-0.03	0.01	0.40*	0.21*

RSA = relationship satisfaction; AAX = attachment anxiety; AAV = attachment avoidance; JEA = jealousy; TRU = trust; SEL = self-esteem; MUT = mutuality; EMC = emotional control; DIF = differentiation; SXS = sexual satisfaction; SAT = sociosexual attitude; SDE = sociosexual desire; SBE = sociosexual behavior. $^{*}p < .05$.

Measurement Invariance Analyses

Table S2. Measurement Invariance Analysis of the RAS Across Gender.

Structural equation modeling was used for analysis. CFI = comparative fit index; RMSEA = root-mean-square error of approximation; SRMR = standardized root-mean-square residual. Group sizes were equal at 300 each. The base model was estimated with a mean structure. Factorial structure (configural), factor loadings (metric), and intercepts (scalar) were progressively restricted. Invariance was indicated by a change of ≥ –.010 in CFI, supplemented by a change of ≤ .015 in RMSEA (1).

Model	χ^2	*df*	$\Delta\chi^2$	Δdf	*p*	CFI	ΔCFI	RMSEA	ΔRMSEA	SRMR
Base	40.932	14				.996		.057		.050
Configural	55.744	28	14.811	14	.391	.996	.000	.058	.001	.054
Metric	79.695	34	23.952	6	< .001	.994	–.002	.067	.009	.061
Scalar	78.408	51	–1.287	17	> .999	.996	.002	.042	–.025	.054

Table S3. Measurement Invariance Analysis of the Subscale Anxiety of the ECR-RD8 Across Gender.

Model	χ^2	*df*	$\Delta\chi^2$	Δdf	*p*	CFI	ΔCFI	RMSEA	ΔRMSEA	SRMR
Base	21.080	2				.952		.163		.039
Configural	21.530	4	1.246	2	.536	.956	.003	.160	–.004	.040
Metric	29.978	7	6.701	3	.082	.949	–.007	.129	–.030	.057
Scalar	43.876	10	14.368	3	.002	.934	–.015	.124	–.006	.065

Structural equation modeling was used for analysis. CFI = comparative fit index; RMSEA = root-mean-square error of approximation; SRMR = standardized root-mean-square residual. Group sizes were equal at 300 each. The base model was estimated with a mean structure. Factorial structure (configural), factor loadings (metric), and intercepts (scalar) were progressively restricted. Invariance was indicated by a change of ≥ –.010 in CFI, supplemented by a change of ≤ .015 in RMSEA (1). The χ^2 column includes robust test statistics, while the χ^2 difference test column is derived from two standard test statistics, making it different from the test statistics in the χ^2 column (2).

Table S4. Measurement Invariance Analysis of the Subscale Avoidance of the ECR-RD8 Across Gender.

Model	χ^2	*df*	$\Delta\chi^2$	Δdf	*p*	CFI	ΔCFI	RMSEA	ΔRMSEA	SRMR
Base	1.316	2				1.000		.000		.011
Configural	1.516	4	0.256	2	.880	1.000	.000	.000	.000	.011
Metric [a]	3.835	6	2.423	2	.298	1.000	.000	.000	.000	.024
Scalar	25.602	10	29.629	4	< .001	.956	–.044	.086	.086	.063

Structural equation modeling was used for analysis. CFI = comparative fit index; RMSEA = root-mean-square error of approximation; SRMR = standardized root-mean-square residual. Group sizes were equal at 300 each. The base model was estimated with a mean structure. Factorial structure (configural), factor loadings (metric), and intercepts (scalar) were progressively restricted. Invariance was indicated by a change of ≥ –.010 in CFI, supplemented by a change of ≤ .015 in RMSEA (1). The χ^2 column includes robust test statistics, while the χ^2 difference test column is derived from two standard test statistics, making it different from the test statistics in the χ^2 column (2).

[a] Partial metric invariance was established by restricting the loadings of item 3 to equal values across groups.

Table S5. Measurement Invariance Analysis of the Subscale Jealousy of the Jealousy Questionnaire Across Gender.

Model	χ^2	*df*	$\Delta\chi^2$	Δdf	*p*	CFI	ΔCFI	RMSEA	ΔRMSEA	SRMR
Base	93.544	34				.971		.063		.031
Configural	132.694	68	38.464	34	.274	.968	–.002	.065	.002	.035
Metric	149.174	77	16.464	9	.058	.965	–.004	.064	–.001	.052
Scalar	167.869	86	19.151	9	.024	.961	–.004	.064	.000	.054

Structural equation modeling was used for analysis. CFI = comparative fit index; RMSEA = root-mean-square error of approximation; SRMR = standardized root-mean-square residual. Group sizes were equal at 300 each. Residuals of item 6 and item 11 were allowed to correlate to improve model fit. The base model was estimated with a mean structure. Factorial structure (configural), factor loadings (metric), and intercepts (scalar) were progressively restricted. Invariance was indicated by a change of ≥ –.010 in CFI, supplemented by a change of ≤ .015 in RMSEA (1). The χ^2 column includes robust test statistics, while the χ^2 difference test column is derived from two standard test statistics, making it different from the test statistics in the χ^2 column (2).

Table S6. Measurement Invariance Analysis of the Subscale Trust of the Jealousy Questionnaire Across Gender.

Model	χ^2	*df*	$\Delta\chi^2$	Δdf	*p*	CFI	ΔCFI	RMSEA	ΔRMSEA	SRMR
Base	34.756	5				.940		.186		.040
Configural	31.149	10	–0.538	5	> .999	.951	.011	.164	–.022	.044
Metric	50.085	14	25.718	4	< .001	.931	–.020	.165	.001	.079
Scalar	65.155	18	16.665	4	.002	.924	–.007	.152	–.013	.082

Structural equation modeling was used for analysis. CFI = comparative fit index; RMSEA = root-mean-square error of approximation; SRMR = standardized root-mean-square residual. Group sizes were equal at 300 each. The base model was estimated with a mean structure. Factorial structure (configural), factor loadings (metric), and intercepts (scalar) were progressively restricted. Invariance was indicated by a change of ≥ –.010 in CFI, supplemented by a change of ≤ .015 in RMSEA (1). The χ^2 column includes robust test statistics, while the χ^2 difference test column is derived from two standard test statistics, making it different from the test statistics in the χ^2 column (2).

Table S7. Measurement Invariance Analysis of the RSES Across Gender.

Model	χ^2	*df*	$\Delta\chi^2$	Δdf	*p*	CFI	ΔCFI	RMSEA	ΔRMSEA	SRMR
Base	250.203	35				.988		.101		.069
Configural	285.004	70	34.801	35	.478	.989	.001	.101	.000	.072
Metric	320.194	79	35.191	9	< .001	.987	–.001	.101	.000	.074
Scalar	308.698	98	–11.496	19	> .999	.989	.002	.085	–.016	.072

Structural equation modeling was used for analysis. CFI = comparative fit index; RMSEA = root-mean-square error of approximation; SRMR = standardized root-mean-square residual. Group sizes were equal at 300 each. The base model was estimated with a mean structure. Factorial structure (configural), factor loadings (metric), and intercepts (scalar) were progressively restricted. Invariance was indicated by a change of ≥ –.010 in CFI, supplemented by a change of ≤ .015 in RMSEA (1).

Table S8. Measurement Invariance Analysis of the Subscale Mutuality of the Relationship Self-Efficacy Scale Across Gender.

Model	χ^2	*df*	$\Delta\chi^2$	Δdf	*p*	CFI	ΔCFI	RMSEA	ΔRMSEA	SRMR
Base	267.884	100				.934		.066		.044
Configural	384.052	200	112.820	100	.180	.929	–.005	.069	.002	.050
Metric	412.138	215	28.130	15	.021	.924	–.005	.069	.000	.069
Scalar	525.874	230	370.510	15	< .001	.892	–.032	.079	.011	.076

Structural equation modeling was used for analysis. CFI = comparative fit index; RMSEA = root-mean-square error of approximation; SRMR = standardized root-mean-square residual. Group sizes were equal at 300 each. Residuals of item 3 and item 19, residuals of item 10 and item 18, residuals of item 12 and item 13, and residuals of item 27 and item 35 were allowed to correlate to improve model fit. The base model was estimated with a mean structure. Factorial structure (configural), factor loadings (metric), and intercepts (scalar) were progressively restricted. Invariance was indicated by a change of ≥ –.010 in CFI, supplemented by a change of ≤ .015 in RMSEA (1). The χ^2 column includes robust test statistics, while the χ^2 difference test column is derived from two standard test statistics, making it different from the test statistics in the χ^2 column (2).

Table S9. Measurement Invariance Analysis of the Subscale Emotional Control of the Relationship Self-Efficacy Scale Across Gender.

Model	χ^2	*df*	$\Delta\chi^2$	Δdf	*p*	CFI	ΔCFI	RMSEA	ΔRMSEA	SRMR
Base	7.823	2				.990		.080		.018
Configural	9.731	4	1.893	2	.388	.990	–.001	.079	–.001	.020
Metric	10.415	7	0.087	3	.993	.994	.005	.045	–.034	.021
Scalar	17.873	10	8.054	3	.045	.988	–.007	.055	.010	.032

Structural equation modeling was used for analysis. CFI = comparative fit index; RMSEA = root-mean-square error of approximation; SRMR = standardized root-mean-square residual. Group sizes were equal at 300 each. The base model was estimated with a mean structure. Factorial structure (configural), factor loadings (metric), and intercepts (scalar) were progressively restricted. Invariance was indicated by a change of ≥ –.010 in CFI, supplemented by a change of ≤ .015 in RMSEA (1). The χ^2 column includes robust test statistics, while the χ^2 difference test column is derived from two standard test statistics, making it different from the test statistics in the χ^2 column (2).

Table S10. Measurement Invariance Analysis of the Subscale Differentiation of the Relationship Self-Efficacy Scale Across Gender.

Model	χ^2	*df*	$\Delta\chi^2$	Δdf	*p*	CFI	ΔCFI	RMSEA	ΔRMSEA	SRMR
Base	37.829	5				.959		.118		.033
Configural	43.719	10	6.011	5	.305	.958	–.001	.119	.002	.035
Metric	49.643	14	6.411	4	.170	.955	–.003	.105	–.015	.050
Scalar	69.256	18	21.264	4	< .001	.940	–.016	.107	.003	.058

Structural equation modeling was used for analysis. CFI = comparative fit index; RMSEA = root-mean-square error of approximation; SRMR = standardized root-mean-square residual. Group sizes were equal at 300 each. The base model was estimated with a mean structure. Factorial structure (configural), factor loadings (metric), and intercepts (scalar) were progressively restricted. Invariance was indicated by a change of ≥ –.010 in CFI, supplemented by a change of ≤ .015 in RMSEA (1). The χ^2 column includes robust test statistics, while the χ^2 difference test column is derived from two standard test statistics, making it different from the test statistics in the χ^2 column (2).

Table S11. Measurement Invariance Analysis of the Subscale Sexual Satisfaction of the FPQ Across Gender.

Model	χ^2	*df*	$\Delta\chi^2$	Δdf	*p*	CFI	ΔCFI	RMSEA	ΔRMSEA	SRMR
Base	89.637	5				.994		.168		.053
Configural	82.463	10	–7.174	5	> .999	.996	.002	.156	–.012	.051
Metric	101.782	14	19.319	4	< .001	.995	–.001	.145	–.011	.056
Scalar	116.353	28	14.571	14	.408	.995	.000	.103	–.042	.051

Structural equation modeling was used for analysis. CFI = comparative fit index; RMSEA = root-mean-square error of approximation; SRMR = standardized root-mean-square residual. Group sizes were equal at 300 each. The base model was estimated with a mean structure. Factorial structure (configural), factor loadings (metric), and intercepts (scalar) were progressively restricted. Invariance was indicated by a change of ≥ –.010 in CFI, supplemented by a change of ≤ .015 in RMSEA (1).

Table S12. Measurement Invariance Analysis of the SOI-R Across Gender.

Model	χ^2	*df*	$\Delta\chi^2$	Δdf	*p*	CFI	ΔCFI	RMSEA	ΔRMSEA	SRMR
Base	59.973	24				.985		.051		.035
Configural	85.530	48	24.067	24	.458	.984	–.001	.051	.000	.041
Metric	113.476	56	25.169	8	.001	.974	–.010	.059	.009	.072
Scalar	143.591	61	29.246	5	< .001	.963	–.011	.068	.009	.076

Structural equation modeling was used for analysis. CFI = comparative fit index; RMSEA = root-mean-square error of approximation; SRMR = standardized root-mean-square residual. Individual subscales of the SOI-R could not be separately tested regarding measurement invariance due to insufficient degrees of freedom. Consequently, a combined model was specified, containing three first-order factors (sociosexual attitude, sociosexual desire, and sociosexual behavior) and one second-order factor (sociosexuality). Group sizes were equal at 300 each. The base model was estimated with a mean structure. Factorial structure (configural), factor loadings (metric), and intercepts (scalar) were progressively restricted. Invariance was indicated by a change of ≥ –.010 in CFI, supplemented by a change of ≤ .015 in RMSEA (1). The χ^2 column includes robust test statistics, while the χ^2 difference test column is derived from two standard test statistics, making it different from the test statistics in the χ^2 column (2).

References

1. Chen FF. Sensitivity of goodness of fit indexes to lack of measurement invariance. Structural Equation Modeling: A Multidisciplinary Journal. 2007 Jul 31;14(3): 464–504.
2. Rosseel Y. lavaan: An R package for structural equation modeling. J Stat Soft. 2012;48(2): 1–36.
